# Structural basis of Q-dependent transcription antitermination

**DOI:** 10.1038/s41467-019-10958-8

**Published:** 2019-07-02

**Authors:** Jing Shi, Xiang Gao, Tongguan Tian, Zhaoyang Yu, Bo Gao, Aijia Wen, Linlin You, Shenghai Chang, Xing Zhang, Yu Zhang, Yu Feng

**Affiliations:** 1 Department of Biophysics, and Department of Pathology of Sir Run Run Shaw Hospital, Zhejiang University School of Medicine, 310058 Hangzhou, China; 2 Key Laboratory of Synthetic Biology, CAS Center for Excellence in Molecular Plant Sciences, Shanghai Institute of Plant Physiology and Ecology, Chinese Academy of Sciences, 200032 Shanghai, China; 3 University of Chinese Academy of Sciences, 100049 Beijing, China; 4 Center of Cryo Electron Microscopy, Zhejiang University School of Medicine, 310058 Hangzhou, China

**Keywords:** Structural biology, Transcription

## Abstract

Bacteriophage Q protein engages σ-dependent paused RNA polymerase (RNAP) by binding to a DNA site embedded in late gene promoter and renders RNAP resistant to termination signals. Here, we report a single-particle cryo-electron microscopy (cryo-EM) structure of an intact Q-engaged arrested complex. The structure reveals key interactions responsible for σ-dependent pause, Q engagement, and Q-mediated transcription antitermination. The structure shows that two Q protomers (Q^I^ and Q^II^) bind to a direct-repeat DNA site and contact distinct elements of the RNA exit channel. Notably, Q^I^ forms a narrow ring inside the RNA exit channel and renders RNAP resistant to termination signals by prohibiting RNA hairpin formation in the RNA exit channel. Because the RNA exit channel is conserved among all multisubunit RNAPs, it is likely to serve as an important contact site for regulators that modify the elongation properties of RNAP in other organisms, as well.

## Abstract (web summary)

Bacteriophage Q protein serves as a model regulator for the study of transcription elongation. Here the authors report a cryo-EM structure of an intact Q-engaged arrested complex, revealing the interactions responsible for σ-dependent pause, Q engagement, and Q-mediated transcription antitermination.

## Introduction

Transcription can be divided into three phases: initiation, elongation, and termination. To initiate transcription at a specific DNA sequence, promoter, bacterial RNA polymerase (RNAP) need to form holoenzyme with σ factor. The principle σ factor, σ^70^ in *Escherichia coli* (*E.coli*), contacts RNAP extensively and mediates sequence-specific interactions with promoter DNA^1–8^. In particular, σ conserved region σR2 contacts a domain of β' subunit known as the clamp helices and mediates sequence-specific interactions with the promoter −10 element, while σ conserved region σR4 contacts the β flap tip helix (FTH) and the β' dock, and mediates sequence-specific interaction with the promoter −35 element.

Bacteriophage Q protein has served as a paradigm for studying regulation of transcription elongation^9^. Through its engagement with RNAP, Q renders RNAP resistant to terminators. Q requires two *cis*-acting elements embedded within phage late gene promoter, P_R’_, to engage RNAP: a −10-like sequence and a Q binding element (QBE). The −10-like sequence is located in the initial transcribed region, resembles the promoter −10 element, and mediates σ-dependent pause^10–12^. Q engages the paused RNAP when bound to the QBE, which is located between the −35 element and −10 element of promoter DNA^13^. A successful recruitment to RNAP of bacteriophage λ Q protein (λQ) requires a third *cis*-acting element, the −35-like sequence, which is absent in the late gene promoters of other lambdoid phages^14^.

Genetic and biochemical studies have elucidated some aspects of Q-dependent antitermination^9^. Nevertheless, a precise mechanistic understanding of the process remains elusive, in part, because of a lack of structural information for Q-engaged complexes^15^. The Q protein of bacteriophage 21 (21Q) was characterized as sharing a conserved mechanism of action with Q proteins from other lambdoid phages^16^. To investigate how Q renders RNAP resistant to terminators, and to visualize sequence-specific interaction of Q with QBE, we determined a crystal structure of 21Q at 1.45 Å resolution and a single-particle cryo-electron microscopy (cryo-EM) structure of an intact 21Q-engaged arrested complex at 4.08 Å resolution. The structures show that two 21Q protomers (Q^I^ and Q^II^) bind to the direct-repeat QBE site. The structures further reveal that Q^I^ forms a ring-like structure inside the RNA exit channel, which only allows the passage of single-stranded RNA. Together, our results suggest that Q renders RNAP resistant to termination signals by prohibiting RNA hairpin formation in the RNA exit channel.

## Results

### 21Q-dependent antitermination is boosted by GreB

**Fig. 1:**
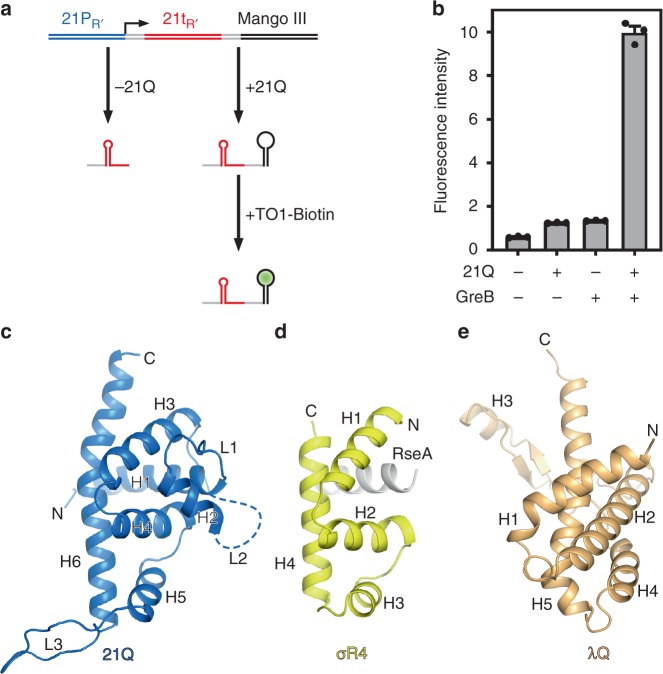
Crystal structure of 21Q. **a** Principle of Mango III transcription antitermination assay. **b** 21Q-dependent antitermination is boosted by GreB (mean ± SEM; 3 determinations). **c** Crystal structure of 21Q. The disordered region of L2 is denoted as a dashed loop. **d** Crystal structure of σR4 in complex with anti-σ RseA (PDB 1OR7 [10.2210/pdb1OR7/pdb]). Yellow, σR4; gray, RseA. **e** Crystal structure of N-terminal truncated λQ (PDB 4MO1 10.2210/pdb4MO1/pdb). Error bars represent mean ± SEM out of *n* = 3 experiments. Source data are provided as a Source Data file

Full length 21Q without any expression tag was purified as soluble protein. To ascertain the antitermination activity of purified 21Q, we developed a transcription antitermination assay by taking advantage of an RNA fluorogenic aptamer, Mango III^17^. Specifically, a DNA fragment, consisting of bacteriophage 21 late gene promoter (21P_R’_) and terminator (21t_R’_) followed by Mango III encoding sequence, is transcribed in vitro (Fig. 1a). If RNAP reads through terminator 21t_R’_, Mango III encoding sequence is transcribed and the transcript becomes fluorescent when bound to TO1-Biotin. The fluorescence intensity in the presence of 21Q is ~2-fold higher than that in the absence of 21Q (Fig. 1b), indicating that purified 21Q improves the read-through of transcription termination signal by RNAP. If GreB is included in the reactions, the fluorescence intensity in the presence of 21Q is ~10-fold higher than that in the absence of 21Q, which is in accordance with the previous reports that Q-dependent antitermination can be boosted by GreB^9,18^.

### Crystal structure of 21Q

The crystal structure of 21Q was determined at 1.45 Å resolution (Fig. 1c and Supplementary Table 1). The structure of 21Q contains six helices (H1-H6) and three long loops (L1-L3). A segment of L2 is disordered in the crystal structure, indicating the intrinsic flexibility of this region. Surprisingly, the structure of 21Q is reminiscent of the binary complex structure of σR4 bound to anti-σ RseA (Fig. 1c, d)^19^. Specifically, the folding of 21Q H3-H6 is similar to the folding of σR4, while 21Q H1 binds to 21Q H3-H6 in the same way as RseA binds to σR4. Because H3 and H4 of σR4 form a helix-turn-helix (HTH) motif and participate in the binding and recognition of the −35 element^20^, the corresponding helices of 21Q (H5 and H6) probably participate in the binding and recognition of the QBE. The crystal structure of N-terminal truncated λQ has been solved previously^21^. Similarly, H4 and H5 of λQ form a HTH motif (Fig. 1e). Other than that, there is no structural similarity between 21Q and λQ, which is consistent with the observation that although both Q proteins act by a similar mechanism, there is no obvious homology to their protein sequences^16^.

### Cryo-EM structure of 21Q-engaged arrested complex

**Fig. 2:**
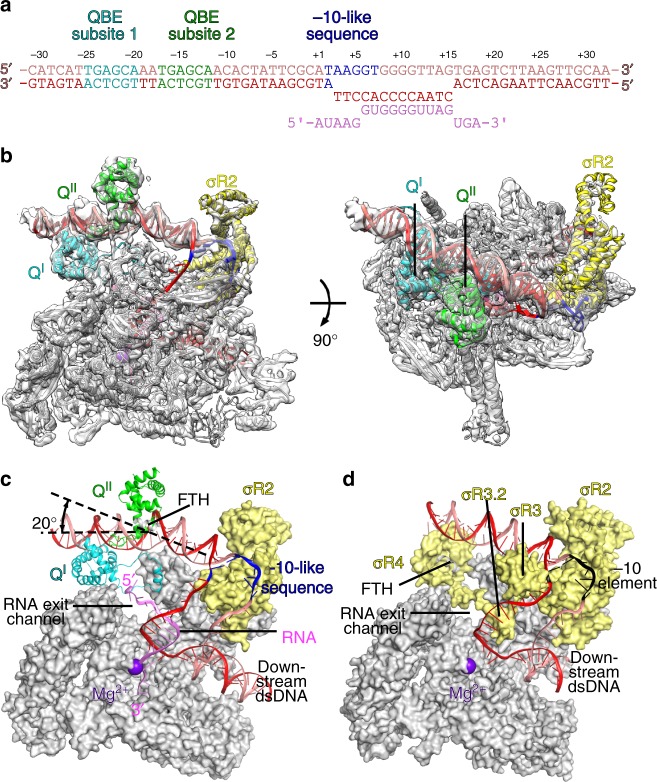
Cryo-EM structure of 21Q-engaged arrested complex. **a** Nucleic-acid scaffold. Only ordered segment in the structure is shown. Salmon, nontemplate strand; red, template strand; pink, RNA; cyan, QBE subsite 1; green, QBE subsite 2; blue, −10-like sequence. Positions are numbered relative to the transcription start site. **b** The cryo-EM density map without B-factor sharpening and the superimposed model of 21Q-engaged arrested complex. Gray, RNAP; cyan, Q^I^; green, Q^II^; yellow, σ^70^; violet, active center Mg^2+^; salmon, nontemplate strand; red, template strand; pink, RNA; blue, −10-like sequence. **c** The model of 21Q-engaged arrested complex. RNAP and σ^70^ are shown as surfaces. View orientation and colors as in the left subpanel of **b**. β, except the FTH, is omitted for clarity. **d** The model of RPo (PDB 4YLN [10.2210/pdb4YLN/pdb]). View orientation and colors as in **c**, except that the promoter -10 element is colored black

To obtain a bona fide 21Q-engaged complex, we first explored whether we could trap the complex through in vitro transcription. The in vitro transcription system included RNAP, 21Q, and a nucleic-acid scaffold corresponding to positions −45 to +34 of 21P_R’_, all C:G base pairs of which between positions +1 and +20 were mutated to G:C base pairs to prevent RNAP from running off the scaffold (positions numbered relative to the transcription start site; Fig. 2a and Supplementary Fig. 1A). As expected, de novo transcription using the scaffold yielded a 20-nt RNA transcript in the absence of CTP (Supplementary Fig. 1B). Cleavage with GreB eliminated the 20-nt RNA transcript and yielded a 14-nt cleavage product, indicating that RNAP is arrested in a backtracked state at position +14. The result of GreB cleavage experiment is consistent with the previous report that Q-engaged RNAP is prone to backtrack to the site of σ-dependent pause where Q initially engages^18^.

Using an analogous method, we prepared the 21Q-engaged arrested complex in the absence of GreB, froze the sample, collected data on Titan Krios, and determined the structure at a nominal resolution of 4.08 Å (Fig. 2b, Supplementary Figs 2–4; Supplementary Table 2). Local resolution calculation indicates that the central core of the structure was determined to 3.4–4.0 Å resolution (Supplementary Fig. 3C). The RNAP of the structure is very similar to the previously reported *E. coli* elongation complex structures^22^, with root-mean-square deviation (RMSD) of 1.02 Å (3000 Cαs aligned).

Consistent with the result of GreB cleavage experiment, RNAP is arrested in a backtracked state at position +14 (Fig. 2a, c, and Supplementary Fig. 4A). Specifically, nucleic-acid scaffold from +3 to +15 is unwound and adopts the same conformation as the transcription bubble in RNAP-promoter open complex (RPo; Fig. 2c, d)^4–8^. Nascent RNA from +6 to+15 forms a 10-bp RNA-DNA hybrid with the template-strand single-stranded DNA (ssDNA), while 5’ 5-nt RNA threads into the RNA exit channel. Although GreB cleavage experiment indicates that 3’ 5-nt RNA is in the secondary channel, only 3-nt RNA is ordered in the structure probably due to weak interactions between the backtracked RNA and the secondary channel.

### Protein–DNA interactions that mediate σ-dependent pause

The structure of 21Q-engaged arrested complex defines the protein–DNA interactions that mediate σ-dependent pause. σ conserved region σR2 interacts with the −10-like sequence in the same way as it interacts with the promoter −10 element in RPo (Fig. 2c, d)^1–8^, consistent with the fact that σR2 is necessary for σ-dependent pause^23^. σ conserved regions σR3, σR3.2, and σR4 are displaced from RNAP probably because of the steric clashes between σR3.2, σR4, and 5’ end of nascent RNA, consistent with the fact that σR3, σR3.2, and σR4 are dispensable for σ-dependent pause^23^.

### Two 21Q protomers engage one RNAP

In the cryo-EM density map, there are two density features adjacent to QBE, which can be attributed to two 21Q protomers (Fig. 2b, c, Supplementary Fig. 4B, C). Protomers bound to the upstream subsite (subsite 1) and the downstream subsite (subsite 2), hereinafter are designated Q^I^ and Q^II^, respectively. Q^I^ has a large conformational change of a short helix, H2, which is detached from the main portion of Q^I^ and moves 30 Å away from its original location (Supplementary Figs 4B, 5). The loops L1 and L2 connecting H2 to the main portion of Q^I^ also move along and form a ring-like structure with H2. Other than that, the rest of Q^I^ is similar to the crystal structure of 21Q, with RMSD of 0.659 Å (119 Cαs aligned). Compared with the crystal structure of 21Q, no obvious conformational change is observed for Q^II^, with RMSD of 0.94 Å (126 Cαs aligned). The two protomers barely interact with each other in 21Q-engaged arrested complex, with a very small buried surface area of 186 Å^2^.

### 21Q-QBE interactions that mediate 21Q engagement

In the structure of 21Q-engaged arrested complex, the upstream double-stranded DNA (dsDNA) adopts roughly the same orientation as in RPo (Fig. 2c, d). Nevertheless, it is bent by 20° at QBE subsite 2 due to a network of protein–protein and protein–DNA interactions (Fig. 2c).

**Fig. 3:**
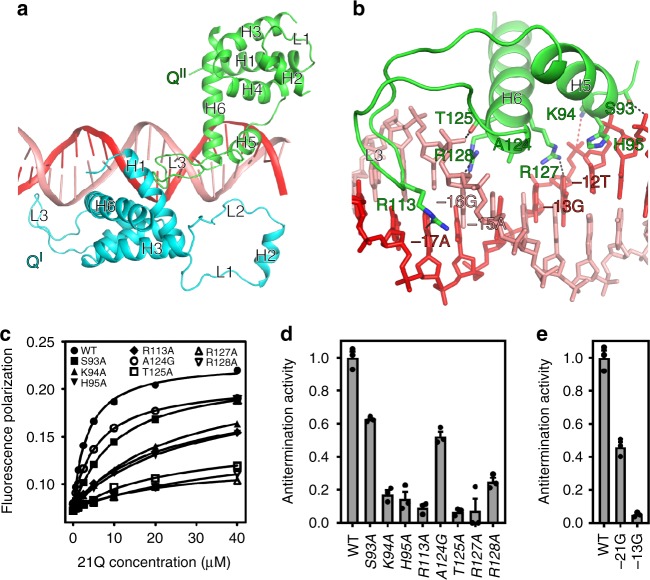
21Q-QBE interactions that mediate 21Q engagement. **a** Q^I^ and Q^II^ contact QBE from different faces of the DNA helix. Cyan, Q^I^; green, Q^II^; salmon, nontemplate strand; red, template strand. **b** Interactions between Q^II^ and QBE subsite 2. Salmon sticks, nontemplate strand; red sticks, template strand; black dashed lines, H-bonds (≤3.5 Å); red dashed line, salt bridge (≤4.5 Å). **c** Effects on 21Q-QBE binding affinity of substitutions of H5, H6, and L3 (mean ± SEM; 3 determinations). **d** Effects on antitermination of substitutions of H5, H6, and L3 (mean ± SEM; 3 determinations). **e** Effects on antitermination of substitutions in QBE subsite 1 (position −21) and subsite 2 (position −13), respectively (mean ± SEM; 3 determinations). Error bars represent mean ± SEM out of *n* = 3 experiments. Source data are provided as a Source Data file

QBE subsite 1 and subsite 2 are direct repeats of 6-bp DNA (TGAGCA; Fig. 2a and Supplementary Fig. 1A). Q^I^ and Q^II^ contact subsite 1 and subsite 2 from different faces of the DNA helix, making similar protein–DNA interactions (Fig. 3a and Supplementary Fig. 6). As expected, the mode of interaction of 21Q with QBE subsites—binding of the second α-helix (H6) of the HTH motif in the DNA major groove—is similar to the mode of interaction of σR4 with −35 element. Specifically, H5 residues S93, K94, and H6 residue T125 are positioned to form two H-bonds and a salt bridge with DNA backbone phosphates, while H5 residue H95, H6 residues A124, R127, and R128 are positioned to make H-bonds and van der Waals interactions with DNA bases that potentially enable sequence readout (Fig. 3b and Supplementary Fig. 4D). In addition, L3, the long loop connecting H5 and H6, inserts into adjacent DNA minor groove with R113 positioned to make a H-bond with a DNA base. Consistent with the structure of 21Q-engaged arrested complex, fluorescence-polarization assay shows that substitution of these residues decreases the binding affinity between 21Q and QBE (Fig. 3c). Moreover, substitution of these residues reduces Q-dependent read-through, verifying their functional importance (Fig. 3d). Furthermore, both substitutions of the C:G base pairs in QBE subsite 1 (position −21) and subsite 2 (position −13) affect Q-dependent transcription antitermination, verifying that both Q^I^-subsite 1 and Q^II^-subsite 2 interactions are essential (Fig. 3e).

The crystal structure of N-terminal truncated λQ shows that it also has a HTH motif analogous to 21Q (Fig. 1e). Genetic and biochemical studies indicate that residues on λQ HTH motif are responsible for QBE recognition^13^. Thus, λQ probably interacts with QBE in a manner similar to 21Q, namely insertion of the second α-helix of the HTH motif in the DNA major groove.

### Q^I^-RNAP interactions that mediate Q^I^ engagement

**Fig. 4:**
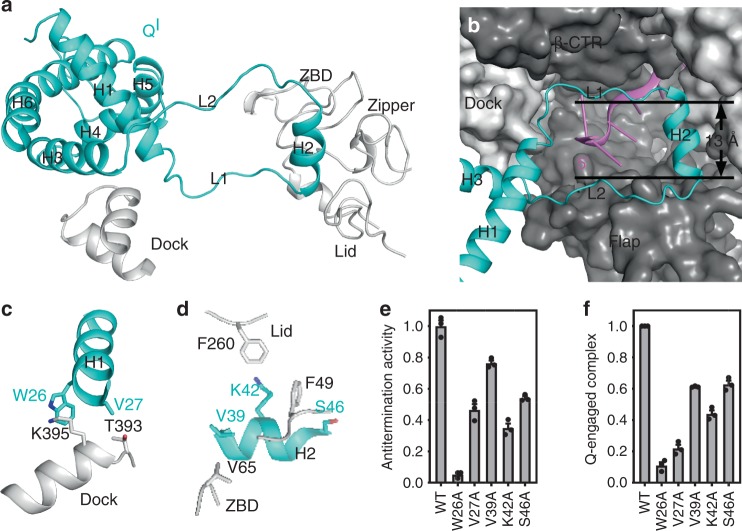
Q^I^-RNAP interactions that mediate Q^I^ engagement. **a** The main portion of Q^I^ interacts with β' dock, while H2 inserts into the RNA exit channel and interacts with the zipper, lid, and ZBD. Cyan, Q^I^; gray, β′. **b** H2, L1, and L2 form a ring-like structure inside the RNA exit channel. RNAP is shown as surface. ZBD is omitted for clarity. Dark gray, β; light gray, β'; cyan, Q^I^; pink, RNA. **c** Interactions between H1 and β' dock. **d** Interactions between H2 and the zipper, lid, and ZBD. **e** Effects on antitermination of substitutions of H1 and H2 (mean ± SEM; 3 determinations). **f** Effects on Q engagement of substitutions of H1 and H2 (mean ± SEM; 4 determinations). Error bars represent mean ± SEM out of *n* = 3 experiments. Source data are provided as a Source Data file

Our structure of 21Q-engaged arrested complex reveals that H2 is detached from the main portion of Q^I^ and forms a ring-like structure with L1 and L2 (Supplementary Figs 4B, 5). The main portion of Q^I^ sits on the exterior opening of the RNA exit channel and interacts with β' dock, while the ring inserts into the RNA exit channel and interacts with the zipper, lid, zinc binding domain (ZBD), flap, and β C-terminal region (β CTR) (Fig. 4a, b). Specifically, H1 residues W26 and V27 are positioned to make van der Waals interactions with dock residues K395 and T393 (Fig. 4c and Supplementary Fig. 4E), while H2 residues V39, K42, and S46 are positioned to make van der Waals interactions with zipper residue F49, lid residue F260, and ZBD residue V65 (Fig. 4d and Supplementary Fig. 4F). A large buried surface area (1384 Å^2^) hints a high-binding affinity between Q^I^ and RNAP. Substitution of the inferred interacting residues decreases 21Q-dependent antitermination (Fig. 4e), confirming the functional importance of the inferred interactions. Taking advantage of the observation that Q-engaged complex moves slower than Q-free complex on native gel, electrophoretic mobility shift assays (EMSA) are performed and Q-engaged complex is quantified. The statistics indicate that substitution of the inferred interacting residues affects Q engagement and DNA interaction (Fig. 4f). However, we can not rule out the possibility that substitution of the inferred interacting residues may affect ring formation, as well.

### Q^II^-RNAP interaction that mediates Q^II^ engagement

**Fig. 5:**
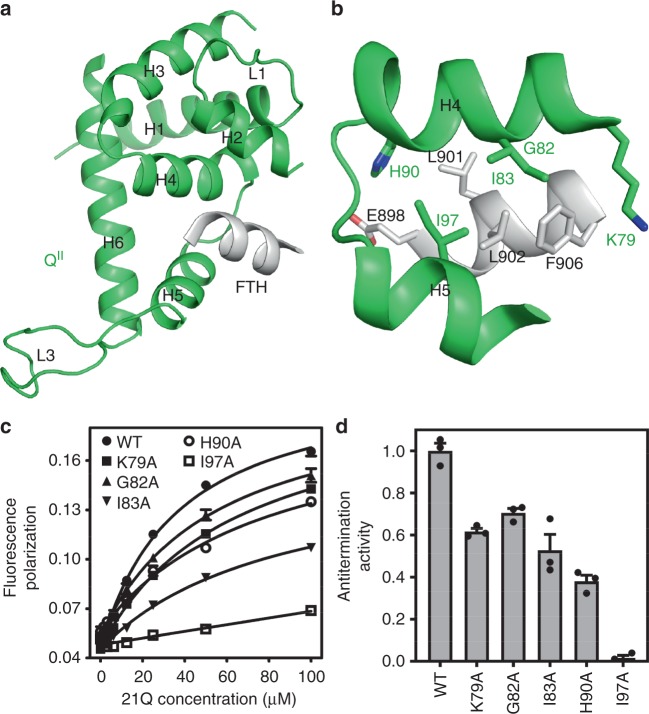
Q^II^-RNAP interaction that mediates Q^II^ engagement. **a** The FTH binds to a groove on Q^II^. Green, Q^II^; gray, FTH. **b** Interactions between H4, H5 and FTH. **c** Effects on 21Q-FTH binding affinity of substitutions of H4 and H5 (mean ± SEM; 3 determinations). **d** Effects on antitermination of substitutions of H4 and H5 (mean ± SEM; 3 determinations). Error bars represent mean ± SEM out of *n* = 3 experiments. Source data are provided as a Source Data file

Taking advantage of the flexibility of the flanking linkers, the FTH moves 30 Å relative to its position in RPo and binds to a groove on Q^II^ (Fig. 5a and Supplementary Fig. 7). In particular, H4 residues K79, G82, I83, H90, and H5 residue I97 are positioned to make van der Waals interactions with FTH residues E898, L901, L902, and F906 (Fig. 5b). Q^II^-RNAP interaction buries a smaller surface area (583 Å^2^) than Q^I^-RNAP interaction (1384 Å^2^), which is consistent with the low binding affinity between 21Q and FTH peptide determined by a fluorescence-polarization assay (Fig. 5c, *K*_D_ = 37 μM). Substitution of residues implicated in Q^II^-FTH interaction results in decreased binding affinity and 21Q-dependent antitermination (Fig. 5c, d), indicating that the inferred interactions occur and are important. In accordance, genetic and biochemical studies showed that λQ also established direct contacts with the FTH during the engagement process^24^.

### Absence of interaction between 21Q and σ

It was reported that λQ interacted with σR4, stabilizing the binding of σR4 to the −35-like sequence embedded within the late gene promoter of bacteriophage λ^14^. However, σR4 is disordered in our structure of 21Q-engaged arrested complex, which is consistent with the observation that there is no −35-like sequence in 21P_R’_ (Fig. 2a and Supplementary Fig. 1A).

## Discussion

**Fig. 6:**
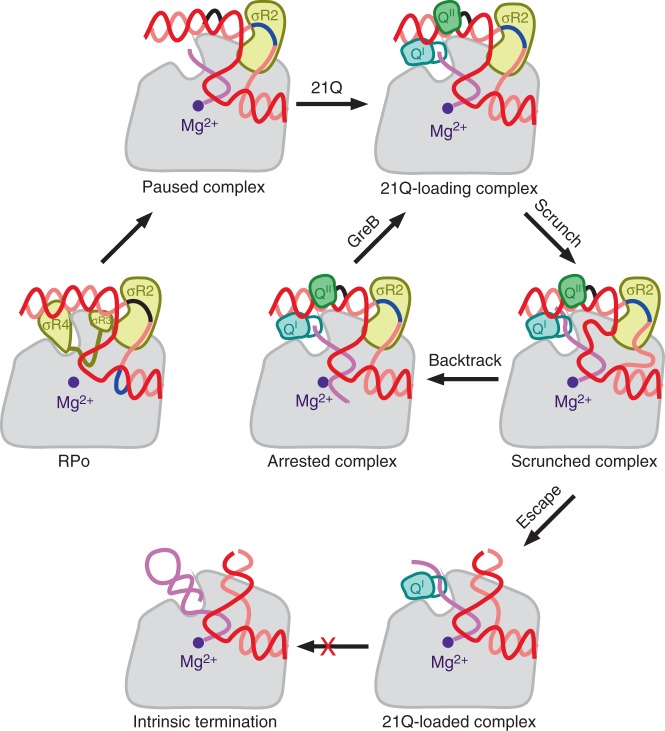
Pathway for formation of a 21Q-dependent termination-resistant elongation complex. Gray, RNAP; cyan, Q^I^; green, Q^II^; yellow, σ^70^; violet, active center Mg^2+^; salmon, nontemplate strand; red, template strand; pink, RNA; black, the promoter −10 element; blue, the −10-like sequence

A pathway for formation of a Q-dependent termination-resistant elongation complex can be drawn based on this work and previous structural and biochemical studies (Fig. 6). During transcription initiation, σR4 and σR2 are anchored to RNAP and make sequence specific contacts with the promoter −35 element and the promoter −10 element, respectively^1–8^. After promoter escape, σR3.2 and σR4 are sequentially displaced because of the steric clashes between σR3.2, σR4, and the nascent RNA, while σR2 is retained^1,25–27^. If a −10-like sequence is encountered, σR2 will make exactly the same contacts with it as in RPo, which will lead to σ-dependent pause^10–12,23^. The interaction between the upstream fork junction of the transcription bubble and σR2 restrains the conformation of upstream dsDNA so that QBE is located in the vicinity of the RNA exit channel. Taking advantage of the pause, Q^I^ and Q^II^ bind to QBE and interact with distinct elements of the RNA exit channel (i.e., the dock, ZBD, zipper, lid, flap, and β CTR for Q^I^; the FTH for Q^II^). Both Q^I^ and Q^II^ contribute to the intricate interactions that stabilize Q-engaged complex and bend the upstream dsDNA by 20°. Since the QBE of the upstream dsDNA and the -10-like sequence of the transcription bubble are tightly anchored to RNAP through Q^I^, Q^II^, and σR2, further extension of the nascent RNA leads to scrunching as in initial transcription^28,29^. If the energy stored in the scrunch is insufficient to disrupt the anchoring, RNAP backtracks into an arrested state analogous to the state in our cryo-EM structure, and cleavage of the backtracked RNA must occur in order to resume transcription^18^. If the energy stored in the scrunch is sufficient to disrupt the anchoring, RNAP escapes from the arrested state and resumes elongation. Considering that the equilibrium dissociation constant for Q^II^-FTH interaction (*K*_D_ = 37 μM) is much higher than the working concentration of 21Q (0.1 μM), Q^II^ probably dissociates after arrest escape. Considering that the buried surface area for Q^I^-RNAP interaction is twice of the buried surface area for Q^II^-RNAP interaction, Q^I^ is probably retained and travels along with RNAP after arrest escape.

A long-standing question has been how Q renders RNAP resistant to intrinsic terminators, which are transcribed to form a GC-rich hairpin followed by a 7–8 nt U-tract^30^. According to the structure of 21Q-engaged arrested complex, the ring-like structure of Q^I^ inserts into the RNA exit channel and serves as a gate (Fig. 4b). Because the side chains of L1 and L2 residues are not modeled due to their weak density map (Supplementary Fig. 4B), the width of the gate cannot be measured. However, it should be less than 13 Å (the shortest distance between Cαs; Fig. 4b), which is too small to accommodate RNA hairpin (diameter > 20 Å)^31,32^. Therefore, we infer that Q^I^ renders RNAP resistant to intrinsic termination by prohibiting RNA hairpin formation in the RNA exit channel.

Cryo-EM structures of protein N, another antiterminator from bacteriophage, in complex with RNAP along with other host factors have been reported^33,34^. Due to its intrinsic disorder, it can thereby adopt a highly elongated conformation, bridge large distances, and interact with RNA, upstream DNA, the hybrid, host factors, and various elements of RNAP that are remote from each other. In contrast with N, 21Q and likely other Q proteins are positioned in a similar locale and only contact elements of the RNA exit channel.

In conclusion, the structure of 21Q-engaged arrested complex defines the mechanisms by which 21Q loads onto and alters the functional properties of elongation complex. The structure demonstrates that the RNA exit channel can serve as a direct target for regulators of transcription elongation. Because the RNA exit channel is a conserved feature of all multisubunit RNAPs^35^, it is likely to serve as an important contact site for regulators that modify the elongation properties of RNAP in other organisms, as well.

## Methods

### Bacteriophage 21 Q protein

*E. coli* strain BL21(DE3) (Invitrogen, Inc.) was transformed with plasmid pET28a-21Q (GENEWIZ, Inc.) encoding 21Q under control of the bacteriophage T7 gene 10 promoter, or with a pET28a-21Q derivative constructed by use of site-directed mutagenesis (QuikChange Site-Directed Mutagenesis Kit; Agilent, Inc.). Single colonies of the resulting transformants were used to inoculate 50 ml LB broth containing 50 μg/ml kanamycin, and cultures were incubated 16 h at 37 °C with shaking. Aliquots (10 ml) were used to inoculate 1 l LB broth containing 50 μg/ml kanamycin, cultures were incubated at 37 °C with shaking until OD_600_ = 0.6, cultures were induced by addition of IPTG to 1 mM, and cultures were incubated 18 h at 16 °C. Then cells were harvested by centrifugation (5000×*g*; 15 min at 4 °C), resuspended in 20 ml buffer A (10 mM Tris-HCl, pH 7.5, 0.2 M NaCl, 5% glycerol, 1 mM EDTA, and 1 mM DTT) and lysed using a JN-02C cell disrupter (JNBIO, Inc.). The lysate was centrifuged (20,000×*g*; 30 min at 4 °C), and the supernatant was loaded onto a 5 ml column of HiTrap Heparin HP (GE Healthcare, Inc.) equilibrated in buffer A and eluted with a 100 ml linear gradient of 0.2–1 M NaCl in buffer A. The sample was further purified by cation-exchange chromatography on a Mono S 10/100 GL column (GE Healthcare, Inc.; 160 ml linear gradient of 0.2–1 M NaCl in buffer A). Fractions containing 21Q were pooled and stored at −80 °C. Yields were ~1–10 mg/L, and purities were >95%.

### E. coli σ^70^

*E. coli* strain BL21(DE3) (Invitrogen, Inc.) was transformed with plasmid pGEMD^36^. Single colonies of the resulting transformants were used to inoculate 50 ml LB broth containing 100 μg/ml ampicillin, and cultures were incubated 16 h at 37 °C with shaking. Aliquots (10 ml) were used to inoculate 1 l LB broth containing 100 μg/ml ampicillin, cultures were incubated at 37 °C with shaking until OD_600_ = 0.6, cultures were induced by addition of IPTG to 1 mM, and cultures were incubated 3 h at 37 °C. Then cells were harvested by centrifugation (5000×*g*; 15 min at 4 °C), resuspended in 20 ml buffer C (40 mM Tris-HCl, pH 7.9, 0.3 M KCl, 10 mM EDTA, and 1 mM DTT) and lysed using a JN-02C cell disrupter (JNBIO, Inc.). After centrifugation (20,000×*g*; 30 min at 4 °C), the pellet was washed with 25 ml buffer C twice, resuspended in buffer D (40 mM Tris-HCl, pH7.9, 6 M GuHCl, 1 mM EDTA, and 10% glycerol), and dialyzed against 3× 1 l buffer E (20 mM Tris-HCl, pH7.9, 0.2 M NaCl, 1 mM EDTA, and 5 mM β-mercaptoethanol) for three times. After centrifugation (20,000*×g*; 30 min at 4 °C), the supernatant was loaded onto a Mono Q 10/100 GL column (GE Healthcare, Inc.) equilibrated in buffer F (10 mM Tris-HCl, pH7.9, 1 mM EDTA, 1 mM DTT, and 5% glycerol) and eluted with a 160 ml linear gradient of 0.3–0.5 M NaCl in buffer F. Fractions containing *E. coli* σ^70^ were pooled and stored at −80 °C. Yield was ~50 mg/L, and purity was >95%.

### *E. coli* RNAP core enzyme

*E. coli* RNAP core enzyme was prepared from *E. coli* strain BL21(DE3) (Invitrogen, Inc.) transformed with plasmid pIA900^37^. Single colonies of the resulting transformants were used to inoculate 50 ml LB broth containing 100 μg/ml ampicillin, and cultures were incubated 16 h at 37 °C with shaking. Aliquots (10 ml) were used to inoculate 1 l LB broth containing 100 μg/ml ampicillin, cultures were incubated at 37 °C with shaking until OD_600_ = 0.6, cultures were induced by addition of IPTG to 1 mM, and cultures were incubated 3 h at 37 °C. Then cells were harvested by centrifugation (5000×*g*; 15 min at 4 °C), resuspended in 20 ml lysis buffer (50 mM Tris-HCl, pH 7.9, 0.2 M NaCl, 2 mM EDTA, 5% glycerol, and 5 mM DTT) and lysed using a JN-02C cell disrupter (JNBIO, Inc.). After poly(ethyleneimine) precipitation and ammonium sulfate precipitation, the pellet was resuspended in buffer G (10 mM Tris-HCl, pH 7.9, 0.5 M NaCl, and 5% glycerol) and loaded onto a 5 ml column of Ni-NTA agarose (Qiagen, Inc.) equilibrated with buffer G. The column was washed with 25 ml buffer G containing 20 mM imidazole and eluted with 25 ml buffer G containing 0.15 M imidazole. The eluate was diluted in buffer F and loaded onto a Mono Q 10/100 GL column (GE Healthcare, Inc.) equilibrated in buffer F and eluted with a 160 ml linear gradient of 0.3–0.5 M NaCl in buffer F. Fractions containing *E. coli* RNAP core enzyme were pooled and stored at −80 °C. Yield was ~2.5 mg/L, and purity was >95%.

### *E. coli* RNAP-σ^70^ holoenzyme

*E. coli* RNAP core enzyme and *E. coli* σ^70^ were incubated in a 1:4 ratio for 1 h at 4 °C. The reaction mixture was applied to a HiLoad 16/600 Superdex 200 column (GE Healthcare, Inc.) equilibrated in 10 mM HEPES, pH 7.5, and 50 mM KCl, and the column was eluted with 120 ml of the same buffer. Fractions containing *E. coli* RNAP-σ^70^ holoenzyme were pooled and stored at −80 °C.

### GreB

*E. coli* strain BL21(DE3) (Invitrogen, Inc.) was transformed with plasmid pMO1.4His encoding N-hexahistidine-tagged GreB under the control of the *trc* promoter^38^. Single colonies of the resulting transformants were used to inoculate 50 ml LB broth containing 100 μg/ml ampicillin, and cultures were incubated 16 h at 37 °C with shaking. Aliquots (10 ml) were used to inoculate 1 l LB broth containing 100 μg/ml ampicillin, cultures were incubated at 37 °C with shaking until OD_600_ = 0.6, cultures were induced by addition of IPTG to 1 mM, and cultures were incubated an additional 3 h at 37 °C. Cells were harvested by centrifugation (5000×*g*; 15 min at 4 °C), resuspended in 20 ml buffer B (40 mM Tris-HCl, pH 7.5, 0.8 M NaCl) and lysed using a JN-02C cell disrupter (JNBIO, Inc.). The lysate was centrifuged (20,000×*g*; 30 min at 4 °C), and the supernatant was loaded onto a 2 ml column of Ni-NTA agarose (Qiagen, Inc.) equilibrated with buffer B. The column was washed with 10 ml buffer B containing 0.25 M imidazole and eluted with 10 ml buffer B containing 0.6 M imidazole. The eluate was concentrated to 2 ml using an Amicon Ultra-15 centrifugal filter (10 kDa MWCO; Merck Millipore, Inc.) and applied to a HiLoad 16/600 Superdex 200 column (GE Healthcare, Inc.) equilibrated in 40 mM Tris-HCl, pH 7.5, 0.8 M NaCl, 1 mM EDTA, and 1 mM DTT, and the column was eluted with 120 ml of the same buffer. Fractions containing GreB were pooled and stored at −80 °C. Yield was ~2.5 mg/L, and purity was >95%.

### Mango III transcription antitermination assay

A DNA fragment corresponding to −148 to +214 of the bacteriophage 21 late gene promoter^16^ followed by Mango III coding sequence^17^ was synthesized and inserted into pUC57 (GENEWIZ, Inc.). The DNA fragment was amplified by PCR, was purified using the QIAquick PCR Purification Kit (Qiagen, Inc.), and was stored at −80 °C. Transcription antitermination assay was performed in a 384-well microplate format. Reaction mixtures contained (50 μl): 0 or 0.1 μM 21Q, 0 or 0.1 μM GreB, 0.1 μM *E. coli* RNAP-σ^70^ holoenzyme, 20 nM DNA fragment, 1 μM TO1-Biotin, 0.2 mM ATP, 0.2 mM UTP, 0.2 mM GTP, 0.2 mM CTP, 50 mM Tris-HCl, pH 8.0, 0.1 M KCl, 10 mM MgCl_2_, 1 mM DTT, and 5% glycerol. Following incubation for 20 min at 37 °C, fluorescence emission intensities were measured using a Varioskan Flash Multimode Reader (ThermoFisher, Inc.; excitation wavelength = 510 nm; emission wavelength = 535 nm). Relative antitermination activities of 21Q derivatives were calculated using:

1 $$A = \left( {I - I_0} \right)/\left( {I_{{\mathrm{WT}}} - I_0} \right)$$

where *I*_WT_ and *I* are the fluorescence intensities in the presence of 21Q and 21Q derivatives, while *I*_0_ is the fluorescence intensity in the absence of 21Q.

### Crystallization and cryo-cooling

Crystallization trails were performed using commercial screening solutions (Hampton Research, Inc. and Qiagen, Inc.) and the hanging-drop vapor diffusion technique (drop: 1 μl protein plus 1 μl screening solution; reservoir: 400 μl screening solution; 23 °C). A total of 300 conditions were screened. Under several conditions, crystals appeared within 1 week. The optimized condition (drop: 1 μl protein plus 1 μl 0.2 M KF and 14% PEG3350; reservoir: 400 μl 0.2 M KF and 14% PEG3350; 23 °C) yielded high-quality, plate-like crystals with dimensions of 0.1 mm × 0.1 mm × 0.05 mm in 1 week. Crystals were transferred to reservoir solution containing 10% (v/v) glycerol and flash-cooled with liquid nitrogen.

### Crystal data collection and structure solution

Diffraction data were collected from cryo-cooled crystals at Shanghai Synchrotron Radiation Facility (SSRF) beamline 17U, processed using HKL2000^39^. The structure was solved by molecular replacement with Molrep^40^ using the structure of σR4 in complex with anti-σ RseA (PDB 1OR7 [10.2210/pdb1OR7/pdb])^19^ as the search model. The model of 21Q was built in Coot^41^ and refined in Phenix^42^.

### Radioactive transcription assay

DNA oligonucleotides (Sangon Biotech, Inc.) were dissolved in nuclease-free water to ~1 mM and stored at −80 °C. Template strand DNA and nontemplate strand DNA were annealed at a 1:1 ratio in 10 mM Tris-HCl, pH 7.9, 0.2 M NaCl and stored at −80 °C. Radioactive transcription assay was performed in reaction mixtures containing (20 μl): 0.1 μM 21Q, 0 or 0.1 μM GreB, 0.1 μM *E. coli* RNAP-σ^70^ holoenzyme, 20 nM DNA scaffold, 50 mM Tris-HCl, pH 8.0, 0.1 M KCl, 10 mM MgCl_2_, 1 mM DTT, and 5% glycerol. Reaction mixtures were incubated 5 min at 37 °C, supplemented with 0.2 mM ATP, 0.2 mM UTP, 0.2 mM GTP, and 0.2 μl 3.3 μM [α-^32^P]UTP (100 Bq/fmol), and RNA synthesis was allowed to proceed for 20 min at 37 °C. Reactions were terminated by adding 10 μl loading buffer (10 mM EDTA, 0.02% bromophenol blue, 0.02% xylene cyanol, and 98% formamide) and boiling for 5 min. Products were applied to 15% urea-polyacrylamide slab gels (19:1 acrylamide/bisacrylamide), electrophoresed in 90 mM Tris-borate, pH 8.0, and 0.2 mM EDTA, and analyzed by storage-phosphor scanning (Typhoon; GE Healthcare, Inc.)

### Assembly of 21Q-engaged arrested complex

21Q-engaged arrested complex was prepared using the same DNA scaffold as the radioactive transcription assay. Reaction mixture contained (5 ml): 0.4 μM 21Q, 0.1 μM *E. coli* RNAP-σ^70^ holoenzyme, 0.11 μM DNA scaffold, 50 mM Tris-HCl, pH 8.0, 0.1 M KCl, 10 mM MgCl_2_, and 1 mM DTT. Reaction mixture was incubated 10 min at 37 °C, supplemented with 1 mM ATP, 1 mM UTP, and 1 mM GTP, and RNA synthesis was allowed to proceed for 10 min at 37 °C. Reaction mixture was concentrated to 13 μM using an Amicon Ultra-0.5 ml centrifugal filter (10 kDa MWCO; Merck Millipore, Inc.).

### Cryo-EM grid preparation

Immediately before freezing, 8 mM CHAPSO was added to the sample. C-flat grids (CF-1.2/1.3–4 C; Protochips, Inc.) were glow-discharged for 60 s at 15 mA prior to the application of 3 μl of the complex, then plunge-frozen in liquid ethane using a Vitrobot (FEI, Inc.) with 95% chamber humidity at 10 °C.

### Cryo-EM data acquisition and processing

The grids were imaged using a 300 kV Titan Krios (FEI, Inc.) equipped with a K2 Summit direct electron detector (Gatan, Inc.). Images were recorded with Serial EM^43^ in counting mode with a physical pixel size of 1.307 Å and a defocus range of 1.5–2.5 μm. Data were collected with a dose of 8 e/pixel/s. Images were recorded with a 12 s exposure and 0.25 s subframes to give a total dose of 56 e/Å^2^. Subframes were aligned and summed using MotionCor2^44^. The contrast transfer function was estimated for each summed image using CTFFIND4^45^. From the summed images, ~10,000 particles were manually picked and subjected to 2D classification in RELION^46^. 2D averages of the best classes were used as templates for auto-picking in RELION. Auto-picked particles were manually inspected, then subjected to 2D classification in RELION. Poorly populated classes were removed, resulting in a dataset of 279,736 particles. These particles were 3D classified in RELION using a map of *E. coli* elongation complex (EMD-8585 [https://www.emdataresource.org/EMD-8585])^22^ low-pass filtered to 40 Å resolution as a reference. 3D classification resulted in 4 classes. Particles in Class 2 were 3D auto-refined, then subjected to 3D classification focused on QBE. From this classification, the best-resolved class containing 64,497 particles was 3D auto-refined and post-processed in RELION.

### Cryo-EM model building and refinement

The model of RNAP from the structure of *E. coli* elongation complex (PDB 6ALF [10.2210/pdb6ALF/pdb])^22^, the models of σR1.2, σR2, and FTH from the structure of *E. coli* RPo (PDB 6CA0 [10.2210/pdb6CA0/pdb])^7^, and the model of 21Q crystal structure were fitted into the cryo-EM density map using Chimera^47^. The model of nucleic acids was built manually in Coot^41^. The coordinates were real-space refined with secondary structure restraints in Phenix^42^.

### Fluorescence polarization assays of 21Q-QBE interaction

5’ 6-FAM labeled template strand DNA oligonucleotide (5’-TGTTGCTCATTTGC-3’, Sangon Biotech, Inc.) and nontemplate strand DNA oligonucleotide (5’-GCAAATGAGCAACA-3’, Sangon Biotech, Inc.) were annealed at a 1:1 ratio in 10 mM Tris-HCl, pH 7.9, 0.2 M NaCl and stored at −80 °C. Equilibrium fluorescence polarization assays were performed in a 96-well microplate format. Reaction mixtures contained (100 μl): 0–40 μM 21Q or 21Q derivative, 0.1 μM 6-FAM-labeled DNA scaffold, 50 mM Tris-HCl, pH 8.0, 0.1 M KCl, 10 mM MgCl_2_, 1 mM DTT, and 5% glycerol. Following incubation mixtures for 10 min at 25 °C, fluorescence emission intensities were measured using a SpectraMax M5 microplate reader (Molecular Devices, Inc.; excitation wavelength = 494 nm; emission wavelength = 518 nm). Fluorescence polarization was calculated using:

2 $$P = (I_{{\mathrm{VV}}} - I_{{\mathrm{VH}}})/(I_{{\mathrm{VV}}} + I_{{\mathrm{VH}}})$$

where *I*_VV_ and *I*_VH_ are fluorescence intensities with the excitation polarizer at the vertical position and the emission polarizer at, respectively, the vertical position and the horizontal position.

Equilibrium dissociation constant, *K*_D_, were extracted by non-linear regression using the equation:

3 $${\it{P}} = {\it{P}}_f + \{ ({\it{P}}_b - {\it{P}}_{\it{f}}){\mathrm{x}}\left[ {\mathrm{T}} \right]/\left( {{\it{K}}_D + \left[ {\mathrm{T}} \right]} \right)\}$$

where *P* is the fluorescence polarization at a given concentration of 21Q, *P*_f_ is the fluorescence polarization for free 6-FAM-labeled DNA scaffold, *P*_b_ is the fluorescence polarization for bound 6-FAM-labeled DNA scaffold, and [T] is the concentration of 21Q or 21Q derivative.

### Electrophoretic mobility shift assay of Q engagement

Electrophoretic mobility shift assays with the DNA scaffold for radioactive transcription assay were performed in reaction mixtures containing (50 μl): 0.1 μM 21Q, 0.1 μM *E. coli* RNAP-σ^70^ holoenzyme, 0.11 μM DNA scaffold, 50 mM Tris-HCl, pH 8.0, 0.1 M KCl, 10 mM MgCl_2_, 1 mM DTT, and 5% glycerol. Reaction mixtures were incubated 10 min at 37 °C, supplemented with 1 mM ATP, 1 mM UTP, and 1 mM GTP, and RNA synthesis was allowed to proceed for 10 min at 37 °C. Reaction mixtures were applied to 5% polyacrylamide slab gels (29:1 acrylamide/bisacrylamide), electrophoresed in 90 mM Tris-borate, pH 8.0, and 0.2 mM EDTA, stained with 4S Red Plus Nucleic Acid Stain (Sangon Biotech, Inc.) according to the procedure of the manufacturer, and analyzed by ImageJ (https://imagej.nih.gov/ij/).

### Fluorescence polarization assays of 21Q-FTH interaction

Equilibrium fluorescence polarization assays of 21Q-FTH interaction were performed analogously to fluorescence polarization assay of 21Q-QBE interaction, using 0–100 μΜ 21Q or 21Q derivative and 0.1 μM N-terminal 5-FAM-labeled peptide (TPEEKLLRAIFGEK, GenScript, Inc.).

### Reporting summary

Further information on research design is available in the Nature Research Reporting Summary linked to this article.

## Supplementary information

Supplementary Information

Peer Review File

Reporting Summary

Source Data

## Data Availability

The data that support the findings of this study are available from the corresponding author upon reasonable request. The accession number for the cryo-EM density map reported in this paper is Electron Microscopy Data Bank: EMD-9852 [https://www.emdataresource.org/EMD-9852]. The accession numbers for the atomic coordinates reported in this paper are Protein Data Bank: 6JNX [https://www.rcsb.org/structure/6JNX] (21Q-engaged arrested complex) and 6JNY [https://www.rcsb.org/structure/6JNY] (21Q). The source data underlying Figs 1B, 3C-E, 4E, 4F, 5C, 5D, and Supplementary Fig. 1B are provided as a Source Data file.
