# Supplementary material for: Structural basis of Q-dependent transcription antitermination
Source: Nat Commun. 2019 Jul 2;10:2925. doi: 10.1038/s41467-019-10958-8 (PMC6606751; doi:10.1038/s41467-019-10958-8)
Supplement: Supplementary file 1 — Supplementary Information [file 41467_2019_10958_MOESM1_ESM.pdf]

# Structural basis of Q-dependent transcription antitermination

Shi et al.

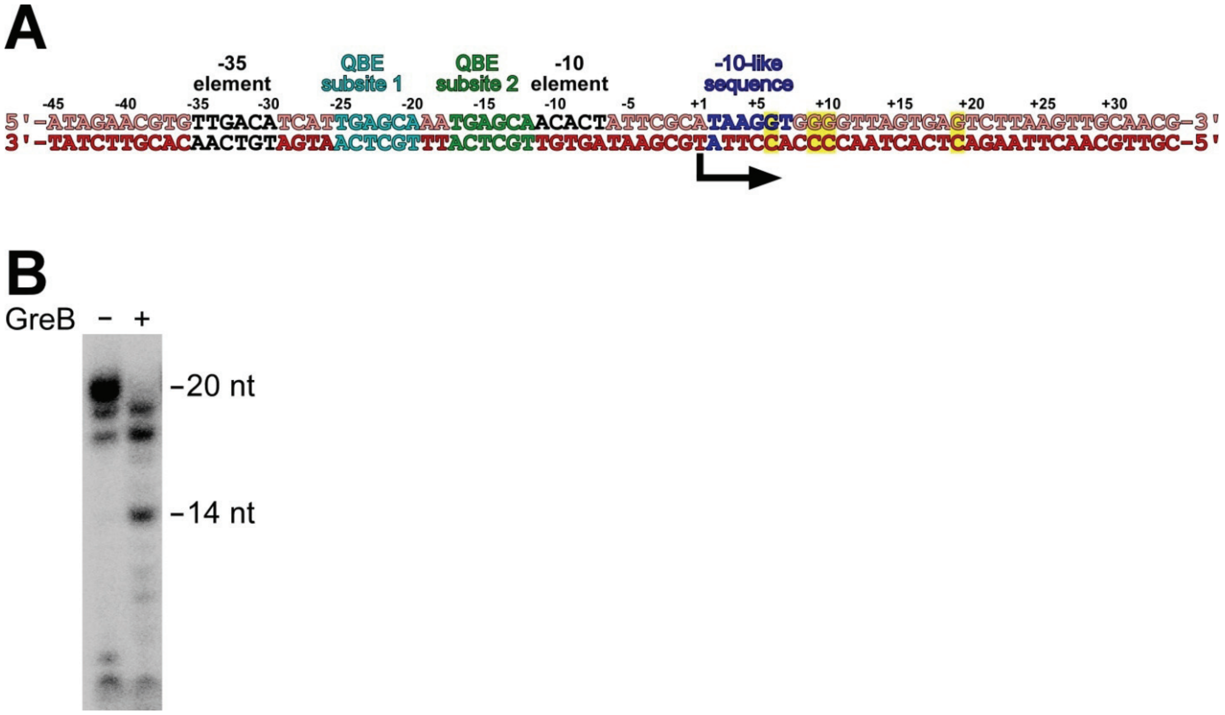


**Supplementary Figure 1. Nucleic acid scaffold.**

(A) Nucleic acid scaffold sequence used for cryo-EM. Salmon, nontemplate strand; red, template strand; yellow, native C:G base pairs are mutated to G:C base pairs. Positions are numbered relative to the transcription start site.

(B) *De novo* transcription with the nucleic acid scaffold in the absence of CTP and in the presence of 21Q.

Source data are provided as a Source Data file.

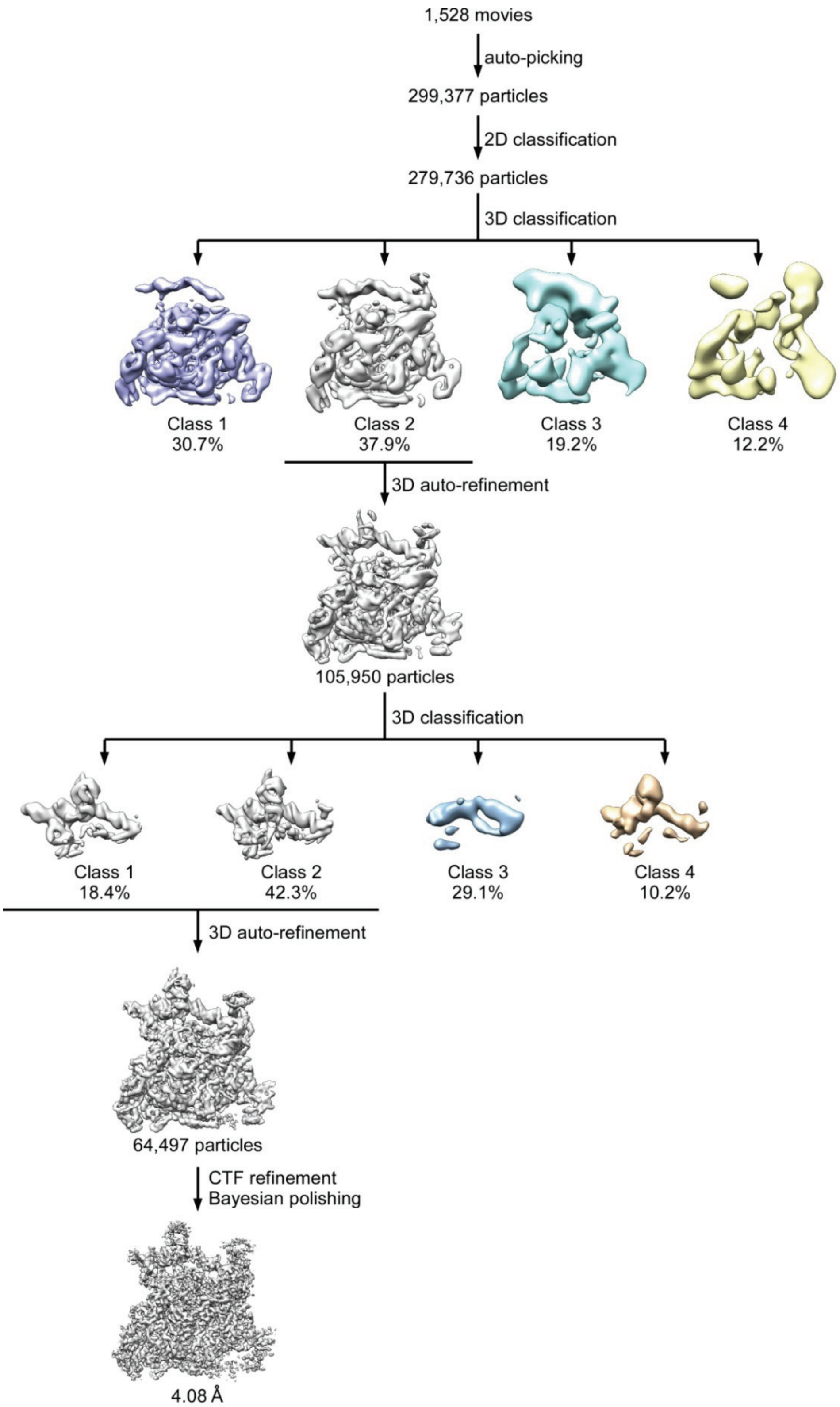


**Supplementary Figure 2. Data processing pipeline for the cryo-EM data.**

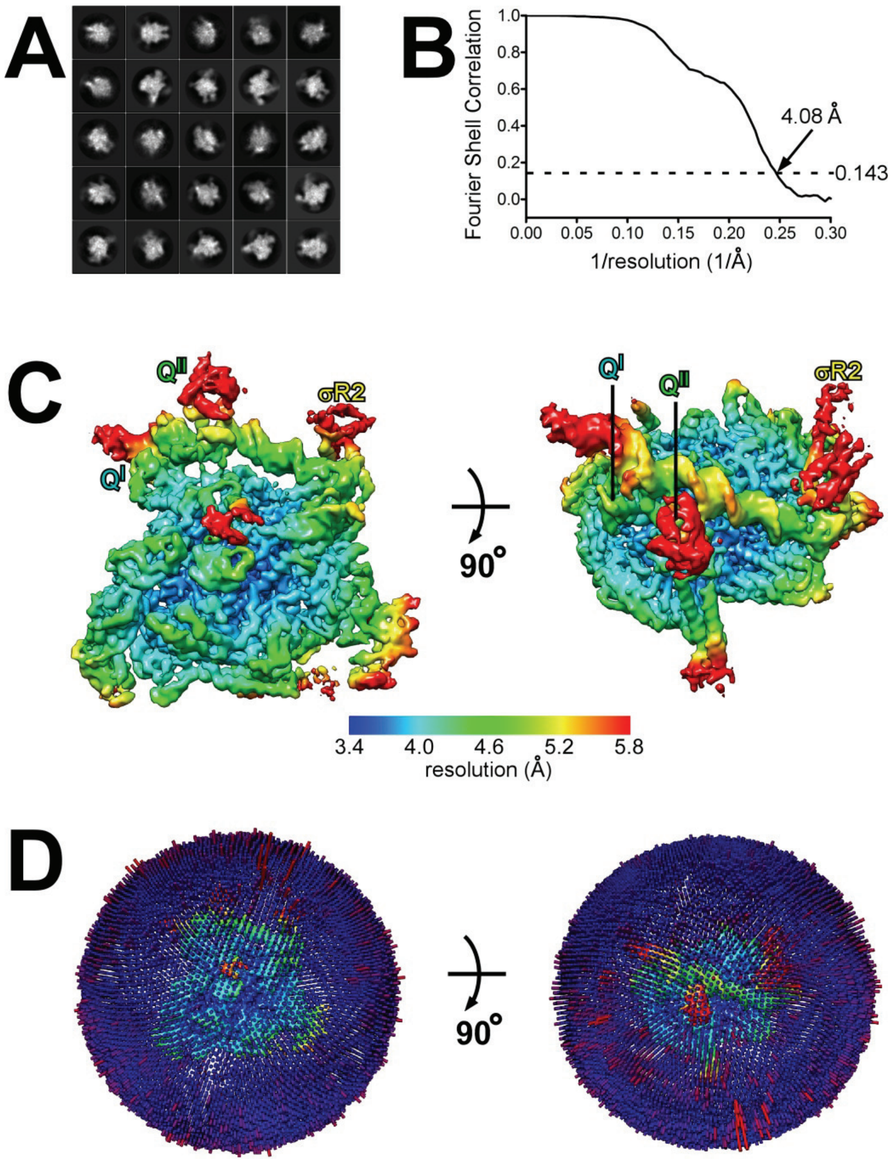


**Supplementary Figure 3. Cryo-EM of 21Q-engaged arrested complex.**

(A) Representative classes from 2D classification.

(B) Gold-standard FSC. The gold-standard FSC was calculated by comparing the two independently determined half-maps from RELION. The dashed line represents the 0.143 FSC cutoff, which indicates a nominal resolution of 4.08 Å.

(C) Cryo-EM density map colored by local resolution. Local resolution calculation was performed using blocres[1]. View orientations as in Figure 2B.

(D) Angular distribution of particle projections. View orientations as in (C).

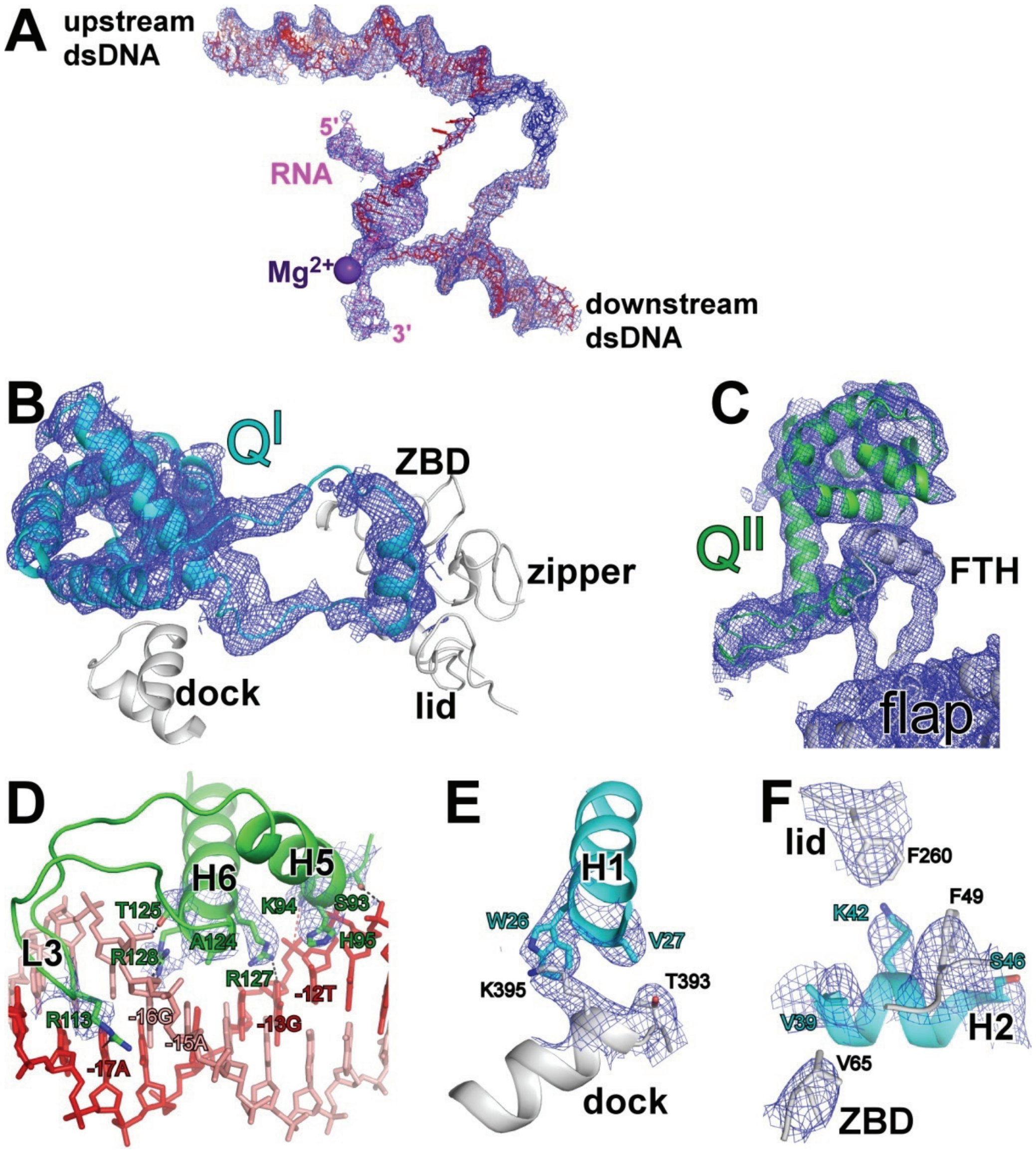


**Supplementary Figure 4. Representative cryo-EM densities and superimposed models.**

(A) Cryo-EM density map (blue mesh) without B-factor sharpening and the superimposed model of the nucleic acids. View orientation and colors as in Figure 2C.

(B) Cryo-EM density map (blue mesh) without B-factor sharpening and the superimposed model of $Q^{I}$. View orientation and colors as in Figure 4A.

(C) Cryo-EM density map (blue mesh) without B-factor sharpening and the superimposed model of $Q^{II}$ and FTH. View orientation and colors as in Figure 5A.

(D) Cryo-EM density map (blue mesh) with B-factor sharpening and the superimposed model for the $Q^{II}$-QBE subsite 2 interactions. View orientation and colors as in Figure 3B.

(E) Cryo-EM density map (blue mesh) with B-factor sharpening and the superimposed model for the H1-dock interactions. View orientation and colors as in Figure 4C.

(F) Cryo-EM density map (blue mesh) with B-factor sharpening and the superimposed model for the H2-β' interactions. View orientation and colors as in Figure 4D.

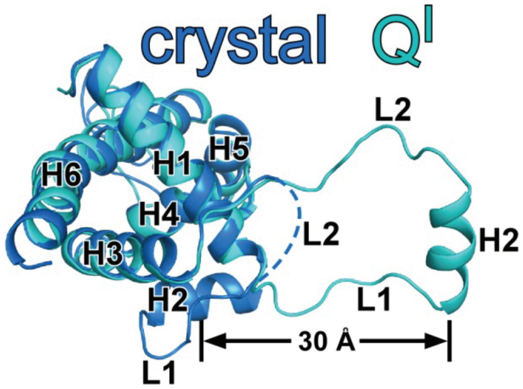


**Supplementary Figure 5. H2 is detached from the main portion of $Q^I$ and forms a ring-like structure with L1 and L2.**

Cyan, $Q^I$ in 21Q-engaged arrested complex; blue, crystal structure of 21Q. The disordered region of L2 is denoted as a dashed loop. View orientation as in Figure 4A.

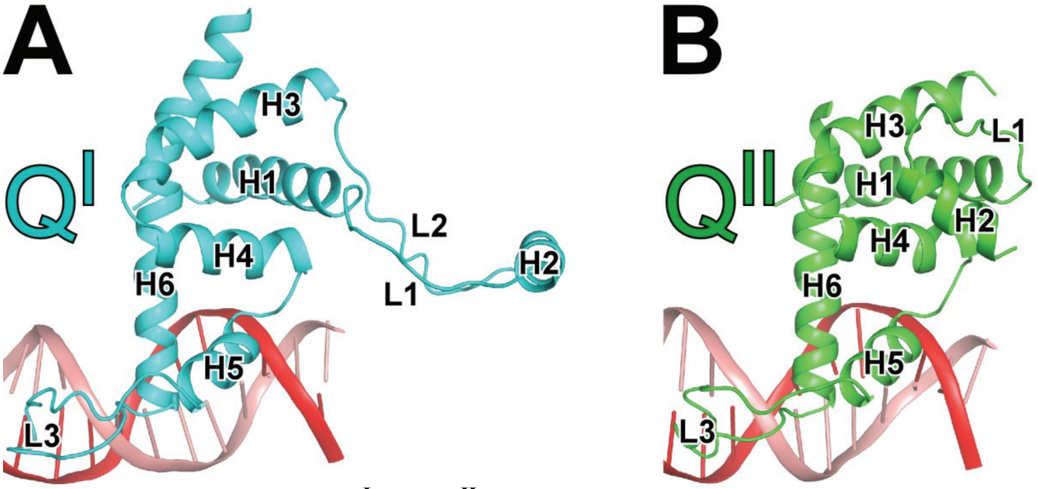


**Supplementary Figure 6. $Q^{I}$ and $Q^{II}$ make similar protein-DNA interactions.**

(A) Interactions between $Q^{I}$ and QBE subsite 1.

(B) Interactions between $Q^{II}$ and QBE subsite 2.

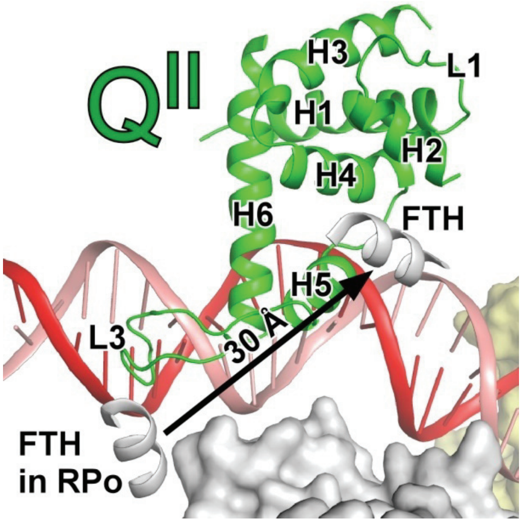


**Supplementary Figure 7. The FTH moves 30 Å relative to its position in RPo and binds to a groove on $Q^{II}$.**

**Supplementary Table 1. Data collection and refinement statistics.**

| | 21Q |
|---|---|
| **Data collection** | |
| Space group | $P2_1$ |
| Cell dimensions | |
| *a*, *b*, *c* (Å) | 32.9, 77.8, 68.7 |
| α, β, γ (°) | 90.0, 98.6, 90.0 |
| Resolution (Å) | 1.45-50.00 (1.45-1.48)* |
| $R_{sym}$ or $R_{merge}$ | 0.073 (0.455) |
| $I / \sigma I$ | 15.8 (3.7) |
| Completeness (%) | 95.5 (85.4) |
| Redundancy | 6.9 (6.5) |
| | |
| **Refinement** | |
| Resolution (Å) | 1.45-31.21 (1.45-1.49) |
| No. reflections | 56,680 |
| $R_{work}$ / $R_{free}$ | 0.21/0.23 (0.25/0.27) |
| No. atoms | |
| Protein | 2275 |
| Ligand/ion | 0 |
| Water | 183 |
| *B*-factors | |
| Protein | 23 |
| Ligand/ion | NA |
| Water | 30 |
| R.m.s. deviations | |
| Bond lengths (Å) | 0.005 |
| Bond angles (°) | 0.735 |

*Values in parentheses are for highest-resolution shell.

**Supplementary Table 2. Cryo-EM data collection, refinement and validation statistics.**

| | 21Q-engaged arrested complex (EMDB-9852) (PDB 6JNX) |
|---|---|
| **Data collection and processing** | |
| Magnification | 22,500 |
| Voltage (kV) | 300 |
| Electron exposure ($e^{-}$/Å$^2$) | 56 |
| Defocus range (μm) | 1.5-2.5 |
| Pixel size (Å) | 1.307 |
| Symmetry imposed | C1 |
| Initial particle images (no.) | 299,377 |
| Final particle images (no.) | 64,497 |
| Map resolution (Å) | 4.08 |
| FSC threshold | 0.143 |
| | |
| **Refinement** | |
| Initial model used (PDB code) | 6ALF |
| Model resolution (Å) | 4.2 |
| FSC threshold | 0.5 |
| Map sharpening *B* factor (Å$^2$) | -123 |
| Model composition | |
| Non-hydrogen atoms | 32,639 |
| Protein residues | 3771 |
| Ligands | 3 |
| *B* factors (Å$^2$) | |
| Protein | 185 |
| Ligand | 141 |
| R.m.s. deviations | |
| Bond lengths (Å) | 0.012 |
| Bond angles (°) | 1.175 |
| Validation | |
| MolProbity score | 2 |
| Clashscore | 9 |
| Poor rotamers (%) | 1 |
| Ramachandran plot | |
| Favored (%) | 92 |
| Allowed (%) | 8 |
| Disallowed (%) | 0 |

**Supplementary References**

1 Cardone, G., Heymann, J.B., & Steven, A.C. One number does not fit all: mapping local variations in resolution in cryo-EM reconstructions. *J. Struct. Biol.* 184, 226-236 (2013).
