# Supplementary material for: Structural basis of Q-dependent transcription antitermination
Source: Nat Commun. 2019 Jul 2;10:2925. doi: 10.1038/s41467-019-10958-8 (PMC6606751; doi:10.1038/s41467-019-10958-8)
Supplement: Supplementary file 2 — Peer Review File [file 41467_2019_10958_MOESM2_ESM.pdf]

Reviewers' comments:

Reviewer #1 (Remarks to the Author):

This is an outstanding piece of work that is technically well done and reveals a fascinating potential mechanism for the phage 21Q antiterminator. I only have a few minor comments:

Major comment: While the role of Q promoter I seems clear, the role of protomer II in the functional mechanism is somewhat murky. What is the role of protomer II?

Minor comments:

1) The authors used the program resmap for the local resolution calculations (Figure S3C). This program is well know to overestimate the resolution from the cryo-EM map. The authors should use the program blocres (Cardone et al., 2013, J. Struct. Biol. 184, 226-236 or the local resolution tools available in cryosparc.

2) Fig. 1C is difficult to understand at least on my print out. The very dark blue of the color makes seeing depth (what is in front of what, etc.) very difficult to see. Maybe a lighter blue color could be used and the figure could be depth cued more steeply to make the image more '3D'?

Reviewer #2 (Remarks to the Author):

Bacteriophage lambda Q protein and other related phage (such as phage 21) Q proteins are one of the best characterized elongation factors for anti-termination complex formation, but the mechanism of preventing transcription termination remains fully elucidated. In this study, authors determined the crystal structure of bacteriophage 21 Q protein (21Q) and also the cryo-EM structure of elongation complex with 21Q to provide structural insight into the 21Q-dependent transcription anti-termination. Authors found that the 21Q structure is similar to the bacterial RNAP sigma factor domain 4, its binds DNA, Q binding element (QBE), and also binds RNAP RNA exit channel to prevent the transcription termination hairpin. Authors carried out structure-based mutagenesis experiments to validate the DNA and RNAP bindings to 21Q for expressing anti-termination activity.

This study provides a structure platform to explain published biochemical and genetic data and also to design further experiments to elucidate the Q-dependent transcription regulation. However, this reviewer identified some problems regarding how to build the model and how to explain the structure-based mutagenesis data as described below. These problems should be addressed before publication.

In vitro transcription assay (Fig. S1) showed that RNAP with Q plus GreB forms three distinct RNA products (14 mer and two more 18 and 19-mers based on their mobilities on gel). Amounts of three RNA products are about the same based on their intensities. The cryo-EM sample was prepared as this in vitro transcription assay condition, indicating that there are three types of elongation complexes at different positions (+14, +18 and +19), but authors claimed that the cryo-EM structure of the Q-engaged elongation complex determined in this study containing only backtracked state at position +14. It is not consistent with the in vitro transcription assay result. Authors ignored other backtracked states at positions +18 and 19 although their amounts are about the same as +14 complex. Is density map clear enough to identified the backtracked state of RNAP? Density map of the nucleic acid shown in Fig. S4 doesn't look clear enough to identify the position of the elongation complex. Is there any additional evidence showing the location of elongation complex?

Authors have to explain how the L1-L2 of QI model in the cryo-EM structure was built. Did authors build the model with side chains? Is density map good enough for building side chains? Fig. S4B doesn't show density corresponding to side chains. In Fig. S8, authors showed the electrostatic potential around L1-L2 of QI. If the model quality around L1-L2 of QI is not good enough, electrostatic potential cannot be predicted.

From the cryo-EM structure, authors found two Q proteins bind at the QBE subsites 1 and 2, and identified amino acid residues involved in the DNA base and backbone interactions. Based on the structure, authors made Q derivates containing amino acid substitutions and characterized them regarding DNA interaction (Fig. 3C). Authors also characterized these Q derivatives regarding anti-termination activities (Fig. 3D) and concluded that the Q and DNA interaction plays an important role in the anti-termination activity. For expressing the anti-termination activity, which Q and DNA interaction (interaction with site 1, site 2 or both) is essential?

In Fig. 4, authors characterized the interaction between QI and RNAP. Based on the cryo-EM structure, authors identified the amino acid residues of Q involved in the RNAP interaction. Q derivatives having amino acid substitutions reduced the Q-dependent anti-termination activity. There are two possibilities to explain this result. First, amino acid substitutions influence the QI and DNA interaction due to disrupting the Q engagement on RNAP. Second, amino acid substitutions influence forming a ring-like structure with L1 and L2. Authors should design an experiment to answer this question.

In Fig. 5, authors showed the QII interacting with the FTH and the density map of QII is shown in Fig. S4C but the density map corresponding to FTH is not shown. The resolution around QII is relatively poor (over 5 Å, Fig. S3C). Authors have to describe evidences used for identifying the QII and FTH interaction.

Minor comments:
In Figure 4, panel B: To measure length of RNA exit path, use surface or CPK representations. a cartoon model shown in this panel represents only main chain but no side chain volume involved in the exit path measurement.

X-ray crystal structure of lamdaQ has been determined, but it was mentioned in this manuscript very briefly (just 2 sentences at the bottom of page 8, no figure). Authors should carry out structural analysis of these Q proteins in detail and describe it with a figure (structural alignment).

Reviewed by Katsuhiko Murakami, Penn State University

Responses to Reviewers' Comments

Reviewer #1 (Remarks to the Author):

This is an outstanding piece of work that is technically well done and reveals a fascinating potential mechanism for the phage 21Q antiterminator. I only have a few minor comments:

We thank the reviewer for the positive assessment of the impact of the work.

Major comment: While the role of Q promoter I seems clear, the role of protomer II in the functional mechanism is somewhat murky. What is the role of protomer II?

Q^{II} helps the engagement of Q^{I} by contributing to the protein-protein and protein-DNA interactions that stabilize Q-engaged complex. This may be the only role of Q^{II}.

Minor comments:

1) The authors used the program resmap for the local resolution calculations (Figure S3C). This program is well known to overestimate the resolution from the cryo-EM map. The authors should use the program blocres (Cardone et al., 2013, J. Struct. Biol. 184, 226-236 or the local resolution tools available in cryosparc.

Local resolution has been recalculated using blocres and Figure S3C has been updated.

2) Fig. 1C is difficult to understand at least on my print out. The very dark blue of the color makes seeing depth (what is in front of what, etc.) very difficult to see. Maybe a lighter blue color could be used and the figure could be depth cued more steeply to make the image more '3D'?

We have updated Figure 1C according to the reviewer's suggestion.

Reviewer #2 (Remarks to the Author):

Bacteriophage lambda Q protein and other related phage (such as phage 21) Q proteins are one of the best characterized elongation factors for anti-termination complex formation, but the mechanism of preventing transcription termination remains fully elucidated. In this study, authors determined the crystal structure of bacteriophage 21 Q protein (21Q) and also the cryo-EM structure of elongation complex with 21Q to provide structural insight into the 21Q-dependent transcription anti-termination. Authors found that the 21Q structure is similar to the bacterial RNAP sigma factor domain 4, its binds DNA, Q binding element (QBE), and also binds RNAP RNA exit channel to prevent the transcription termination hairpin. Authors carried out structure-based mutagenesis experiments to validate the DNA and RNAP bindings to 21Q for expressing anti-termination activity.

This study provides a structure platform to explain published biochemical and genetic data and also to design further experiments to elucidate the Q-dependent transcription regulation. However, this reviewer identified some problems regarding how to build the model and how to explain the structure-based mutagenesis data as described below. These problems should be addressed before publication.

We thank the reviewer for the comments to improve the quality of the work.

In vitro transcription assay (Fig. S1) showed that RNAP with Q plus GreB forms three distinct RNA products (14 mer and two more 18 and 19-mers based on their mobilities on gel). Amounts of three RNA products are about the same based on their intensities. The cryo-EM sample was prepared as this in vitro transcription assay condition, indicating that there are three types of elongation complexes at different positions (+14, +18 and +19), but authors claimed that the cryo-EM structure of the Q-engaged elongation complex determined in this study containing only backtracked state at position +14. It is not consistent with the in vitro transcription assay result. Authors ignored other backtracked states at positions +18 and 19 although their amounts are about the same as +14 complex. Is density map clear enough to identified the backtracked state of RNAP? Density map of the nucleic acid shown in Fig. S4 doesn't look clear enough to identify the position of the elongation complex. Is there any additional evidence showing the location of elongation complex?

In fact, the cryo-EM sample was prepared in the absence of GreB, which has been emphasized on page 6, line 6. *In vitro* transcription assay (Figure S1B) shows that the RNA transcript is more homogenous in the absence of GreB (lane 1) than in the presence of GreB (lane 2). Therefore, the cryo-EM sample was prepared in the absence of GreB, which should give a major transcript of 20-nt as in lane 1. Cleavage with GreB eliminated the 20-nt RNA transcript and yielded a 14-nt cleavage product, indicating that RNAP is arrested in a backtracked state at position +14, which is consistent with the previous literature (Strobel and Roberts, 2014) and the cryo-EM density map.

Authors have to explain how the L1-L2 of QI model in the cryo-EM structure was built. Did authors build the model with side chains? Is density map good enough for building side chains? Fig. S4B doesn't show density corresponding to side chains. In Fig. S8, authors showed the electrostatic potential around L1-L2 of QI. If the model quality around L1-L2 of QI is not good enough, electrostatic potential cannot be predicted.

After checking the cryo-EM density map carefully, we have decided to trim the side chains of L1 and L2 residues and deleted Figure S8.

From the cryo-EM structure, authors found two Q proteins bind at the QBE subsites 1 and 2, and identified amino acid residues involved in the DNA base and backbone interactions. Based on the structure, authors made Q derivates containing amino acid substitutions and characterized them regarding DNA interaction (Fig. 3C). Authors also characterized these Q derivatives regarding anti-termination activities (Fig. 3D) and concluded that the Q and DNA

interaction plays an important role in the anti-termination activity. For expressing the anti-termination activity, which Q and DNA interaction (interaction with site 1, site 2 or both) is essential?

To address the reviewer's question directly, we substitute the C:G base pairs in QBE subsite 1 (position -21) and subsite 2 (position -13), respectively. Both substitutions affect Q-dependent transcription antitermination, verifying that both Q^{I}-subsite 1 and Q^{II}-subsite 2 interactions are essential. The results have been added as Figure 3E and the conclusion has been added to page 8, line 18.

In Fig. 4, authors characterized the interaction between QI and RNAP. Based on the cryo-EM structure, authors identified the amino acid residues of Q involved in the RNAP interaction. Q derivatives having amino acid substitutions reduced the Q-dependent anti-termination activity. There are two possibilities to explain this result. First, amino acid substitutions influence the QI and DNA interaction due to disrupting the Q engagement on RNAP. Second, amino acid substitutions influence forming a ring-like structure with L1 and L2. Authors should design an experiment to answer this question.

Taking advantage of the observation that Q-engaged complex moves slower than Q-free complex on native gel, electrophoretic mobility shift assays (EMSA) have been performed and Q-engaged complex has been quantified. The statistics indicate that the substitution of Q^{I}-RNAP interacting residues affects Q engagement and DNA interaction. However, we can not rule out the possibility that the substitution of Q^{I}-RNAP interacting residues may affect ring formation, as well. The procedure of EMSA has been described on page 19, line 22. The statistics have been added as Figure 4F and the conclusion has been added to page 9, line 17.

In Fig. 5, authors showed the QII interacting with the FTH and the density map of QII is shown in Fig. S4C but the density map corresponding to FTH is not shown. The resolution around QII is relatively poor (over 5 Å, Fig. S3C). Authors have to describe evidences used for identifying the QII and FTH interaction.

The density map of FTH is strong and has been added into Figure S4C. As described in the MATERIALS AND METHODS, the model of FTH from the structure of *E. coli* RPo (PDB 6CA0) was fitted into the density map as a rigid body and refined with secondary structure restrains.

Minor comments:
In Figure 4, panel B: To measure length of RNA exit path, use surface or CPK representations. a cartoon model shown in this panel represents only main chain but no side chain volume involved in the exit path measurement.

After checking the cryo-EM density map carefully, we have decided to trim the side chains of L1 and L2 residues. We have added the following statement on page12, line 1.

"Because the side chains of L1 and L2 residues are not modeled due to their weak density map (Figure S4B), the width of the gate can not be measured. However, it should be less than 13 Å (the shortest distance between Cαs; Figure 4B), which is too small to accommodate RNA hairpin (diameter > 20 Å) [31,32]."

X-ray crystal structure of lamdaQ has been determined, but it was mentioned in this manuscript very briefly (just 2 sentences at the bottom of page 8, no figure). Authors should carry out structural analysis of these Q proteins in detail and describe it with a figure (structural alignment).

As the reviewer suggests, the crystal structure of λQ has been added as Figure 1E. We have added the following statement on page 5, line 11.

"The crystal structure of N-terminal truncated λQ has been solved previously [21]. Similarly, H4 and H5 of λQ form a HTH motif (Figure 1E). Other than that, there is no structural similarity between 21Q and λQ, which is consistent with the observation that although both Q proteins act by a similar mechanism, there is no obvious homology to their protein sequences [16]."

REVIEWERS' COMMENTS:

Reviewer #2 (Remarks to the Author):

Authors responded almost all of my critiques adequately except the QII and FTH interaction. To clarify it, I suggest to modify Fig. S4C showing not only the FTH but also the flap and linkers connecting between the flap and FTH (both the final model and density map) to identify the alpha helix binding on the QII is from the RNAP flap. After this minor modification, I highly recommend to publish this work.
